# A Non‐Pharmacological Perspective to Memory Improvement in Dementia

**DOI:** 10.1002/alz.092480

**Published:** 2025-01-09

**Authors:** Niharika Dadoo, Ajay Prakash, Bikash Medhi

**Affiliations:** ^1^ Postgraduate Institute of Medical Education and Research (PGIMER), Chandigarh, Chandigarh India

## Abstract

**Background:**

Dementia is a progressive clinical syndrome which is marked by pervasive cognitive impairment and deterioration. With the ever‐increasing number of people living with dementia, it has become a global concern. Current medications focus on slowing the progression of dementia and managing the comorbidities and not on directly enhancing memory. Cholinesterase inhibitor drugs like donepezil are prescribed to enhance neurotransmitter function and regulate brain cell activity, thus reducing cognitive decline but they leave memory unaddressed.

A combination of psychology and pharmacology can lead to a more holistic approach to addressing dementia. Newer methods like art therapy can be used in adjunct to pharmacotherapy. Art therapy is a nonverbal creative approach to work around cognitive, memory, motor, emotional and social concerns in dementia. Cultural arts interventions (dance, visual arts, storytelling/theatre, music, and art) can expand the personhood and quality of life of those experiencing dementia. According to Kinney & Rentz (2005), participation in creative arts enhances the social and psychological well‐being of people with a diagnosis of dementia and their caregivers. Drawing, painting collaging and colouring can enhance memory and create an overall sense of wellbeing. Making visual art improves neuroplasticity and the connections between different brain regions. Creative arts programs in the community set‐up can prove to be a non‐stigmatising environment to engage the individuals, their caregivers and the community.

**Conclusion:**

Community‐driven art programs can empower social bonds and people with the tools to contribute to supporting those living with dementia. More research is required on how art and psychotherapy can work in adjunct with pharmacotherapy in reducing the symptoms of dementia.

